# Removal of Ni(II) Ions by Poly(Vinyl Alcohol)/Al_2_O_3_ Nanocomposite Film via Laser Ablation in Liquid

**DOI:** 10.3390/membranes12070660

**Published:** 2022-06-27

**Authors:** Fatemah H. Alkallas, Hoda A. Ahmed, Tahani A. Alrebdi, Rami Adel Pashameah, Salhah H. Alrefaee, Emaan Alsubhe, Amira Ben Gouider Trabelsi, Ayman M. Mostafa, Eman A. Mwafy

**Affiliations:** 1 Department of Physics, College of Science, Princess Nourah bint Abdulrahman University, P.O. Box 84428, Riyadh 11671, Saudi Arabia; fhalkallas@pnu.edu.sa (F.H.A.); taalrebdi@pnu.edu.sa (T.A.A.); aatrabelsi@pnu.edu.sa (A.B.G.T.); 2 Department of Chemistry, Faculty of Science, Cairo University, Cairo 12613, Egypt; ahoda@sci.cu.edu.eg; 3 Chemistry Department, College of Sciences, Taibah University, Yanbu 30799, Saudi Arabia; srfaay@taibahu.edu.sa; 4 Department of Chemistry, Faculty of Applied Science, Umm Al-Qura University, Makkah 24230, Saudi Arabia; rapasha@uqu.edu.sa; 5 Physics Department, Faculty of Science, Taibah University, Yanbu 30799, Saudi Arabia; esobhe@taibahu.edu.sa; 6 Spectroscopy Department, Physics Division Institute, National Research Centre, 33 El Bohouth St. (Former El Tahrir st.), Dokki, Giza 12622, Egypt; 7 Laser Technology Unit, Center of Excellent for Advanced Science, National Research Centre, 33 El Bohouth st. (Former El Tahrir St.), Dokki, Giza 12622, Egypt; emanmwafynrc@gmail.com; 8 Physical Chemistry Department, Advanced Materials Technology and Mineral Resources Research Institute, National Research Centre, Giza 12622, Egypt

**Keywords:** PLAL, Al_2_O_3_, NPs, Nd:YAG, laser ablation, nanocomposite

## Abstract

Al_2_O_3_-poly(vinyl alcohol) nanocomposite (Al_2_O_3_-PVA nanocomposite) was generated in a single step using an *eco*-friendly method based on the pulsed laser ablation approach immersed in PVA solution to be applicable for the removal of Ni(II) from aqueous solution, followed by making a physicochemical characterization by SEM, XRD, FT-IR, and EDX. After that, the effect of adsorption parameters, such as pH, contact time, initial concentration of Ni(II), and medium temperature, were investigated for removal Ni(II) ions. The results showed that the adsorption was increased when pH was 5.3, and the process was initially relatively quick, with maximum adsorption detected within 90 min of contact time with the endothermic sorption process. Moreover, the pseudo-second-order rate kinetics (k_2_ = 9.9 × 10^−4^ g mg^−1^ min^−1^) exhibited greater agreement than that of the pseudo-first-order. For that, the Ni(II) was effectively collected by Al_2_O_3_-PVA nanocomposite prepared by an *eco*-friendly and simple method for the production of clean water to protect public health.

## 1. Introduction

Nano adsorbent materials have sparked tremendous attention in recent years because of their large specific surface area, regular pore structure, and highly controlled surface characteristics. Almost the majority of the adsorbents that are created today to overcome the hazard of heavy metal ions and dyes depend on the engagement of the hazardous chemical compounds with the presence of functional groups on the adsorbents’ surfaces [1,2,3,4,5]. As a result, the matrix’s vast surface area and many adsorption sites are the most critical elements influencing the adsorbent’s adsorption capacity. From hazardous chemical compounds, nickel waste is extremely dangerous to people, ecosystems, and animals. It has been found in a wide range of industrial wastes, including nickel–cadmium batteries, organic compounds, and insecticides [6,7,8,9]. The abundance of nickel ions in the water supply has been linked to a variety of major health issues, including carcinogenic agents. As a result, removing nickel from wastewater and industrial pollutants is critical [10,11,12,13]. Several techniques have been employed to remove heavy metals from wastewater, including chemical precipitation, ion-exchange sorption, and solvent extraction. One of these methods, the adsorption method has been frequently employed because of its low cost and great efficacy [14,15,16,17,18,19].

Aluminum oxide nanostructured material (Al_2_O_3_) has been studied as a promising adsorbent for heavy metal ion elimination because of its large surface area. Moreover, in the water treatment field, it exhibits better catalytic activity in a variety of organic processes [20,21]. However, in the field of water treatment, powdered material is not suited for recycling. Furthermore, the separation of nanostructured materials from aqueous solutions is quite challenging, especially when the particle size is on the nano-scale. Researchers are currently concentrating on methods of employing nano-powder metal oxides to remove heavy metal ions [22,23,24]. One method is to incorporate nanoparticles into an organic substance, which could be represented by an optimum solution such as a polymer matrix structure. Adsorbent features such as surface area and functional groups on the adsorbent surface should be addressed for the adsorbent to eliminate heavy metal ions. The important qualities of the adsorbent, such as high mechanical and thermal stability and high sorption capacity, should be carefully addressed. For these reasons, selecting appropriate materials for heavy metal ion sorption from an aqueous medium is critical. Adsorbents such as zeolites, polymers, metal oxides, and polymer/metal oxides have been used as materials for the sorption of heavy metal ions. From these promising absorbents, poly (vinyl alcohol) PVA is identified to be associated with various organic and inorganic compounds able to supply hydroxyl groups on its surface for the elimination of heavy metal ions. Because of its unique features such as high porosity, large surface area, and tiny pore size, it has been widely employed for heavy metal ion adsorption. Furthermore, it provides a greater possibility for nanoparticle loading without affecting mechanical stability [25,26,27].

Metal oxide nanoparticles placed inside a polymer matrix have recently piqued the interest of researchers due to the unique features of hybrid nanocomposites. Many factors influence the properties of the created nanocomposites, including the synthesis method, its morphological structure, the characteristics of the metal oxide nanoparticles that are implemented in PVA, and the physicochemical properties of both metal oxide and polymer molecules in the interaction [28,29,30]. One of these techniques, that helped us to vary the characteristics of embedding metal oxide in a simple way, is the pulsed laser ablation in liquid media approach. It is a simple, quick, economical, green, dependable method for producing a wide range of nanostructured materials and a commonly used technology for producing nanocomposite adsorbent materials that has received a lot of attention recently. This method allows us to produce nanostructured materials in high purity without any minor products as it is based on using just required precursor pure metals not in their salts at the beginning of the preparation, and it did not require surfactants to preserve the size in nanoscale form. Furthermore, the normal behavior of the preparation of nanostructured materials via any physical or chemical approach methods is associated with the generation of hazard vapor fumes consisting of very finer powders from the prepared nanostructured form, which have a very dangerous side effect on the environment and human health as they are capable of passing through human skin, while in this method, all the preparation steps were carried out inside a liquid medium, which prevents the transfer of any vapor fumes to the environment or human bodies. Therefore, this method could be considered an *eco*-friendly and green method. Furthermore, this method is eligible to form nanocomposite structures by ablating many metal targets at the same time or changing the liquid media with a matrix structure, such as PVA or CNTs. Therefore, this method fits the need for versatility, which affects the shape and size of the produced nanostructured materials in comparison to other methods. However, this mass production of this method could represent the main issue faced by the enhancement and development of this method [31,32,33,34,35]. The achievement of the PLAL method in the preparation of Al_2_O_3_ NPs could be summarized as follows: In 2010, S. Khan et al. studied the effect of the ablation time of the continuous wave (CW) of fiber laser to obtain Al_2_O_3_ NPs with different particle sizes [36]. In 2012, V. Piriyawong et al. prepared Al_2_O_3_ NPs by ablating Al pellet immersed in deionized water. It was shown that their particle size could be changed by varying the ablation energy [37]. In 2020, A. Riahi et al. showed the prepared Al_2_O_3_ NPs by laser ablation (7 ns, 1064 nm, and 120 mJ) of Al target immersed in deionized water with have high thermal conductivity [38]. Moreover, M. Eskandari et al. prepared Au@Al_2_O_3_ core-shell in just one step by ablating bulk aluminum deposited with a thin layer from the Au layer immersed in ethanol solution by CW fiber laser (1064 and 40 W). This preparation method could be represented the development of the PLAL method for generating core/shell nanocomposite structures [39]. Furthermore, A. Mostafa et al. showed the capability for preparing different metal oxides embedded in PVA structure in just a one-pot method by ablating metal plates (Al, Cd, and Cu) in PVA solution [40]. Moreover, A. Mostafa et al. showed another facility laser parameter to enhance the PLAL method, by making laser irradiation after the laser ablation process to decrease the particle size of the prepared Al_2_O_3_ NPs [41]. Furthermore, the previous contributions of Al_2_O_3_-PVA nanocomposite for the removal of heavy metals or degraded organic pollutants can be summarized in Table 1.

In this work, an Al_2_O_3_-PVA nanocomposite was effectively produced in just one step using the *eco*-friendly promising tools of the PLAL method to be suitable for nickel ion adsorption from aqueous solutions. The properties of the synthesized hybrid structure were carried out by different techniques. After that, the influence of several factors on the sorption process, such as concentration, contact duration, beginning concentration, and temperature was examined for Ni(II).

## 2. Materials and Experimental Work

### 2.1. Materials

Ultra-pure water was used to make all of the reagents. Poly vinyl alcohol solution (PVA) was purchased from La-113 laboratory, Rasayan, Egypt. Aluminum sheet (Al) with dimension 2 × 2 cm^2^ and thickness of 0.1 mm was purchased from the BDH Chemical Ltd. pool, England. Nickel nitrate (NiNO_3_) was purchased from LOBA Chemie, Laboratory Reagents and fine Chemicals, India, which was used to make a stock solution of Ni(II) ions. Sodium hydroxide (NaOH) and hydrochloric acid (HCl) were purchased from El Nasr Pharmaceutical Co., Giza, Egypt.

### 2.2. Preparation of PVA Solution

A 10 wt.% PVA was produced by dissolving 10 g of PVA in 100 mL of ultra-pure water and maintaining it at 80 °C for 300 min with continued magnetic stirring.

### 2.3. Preparation of Al_2_O_3_-PVA Composite

**Figure 1:**
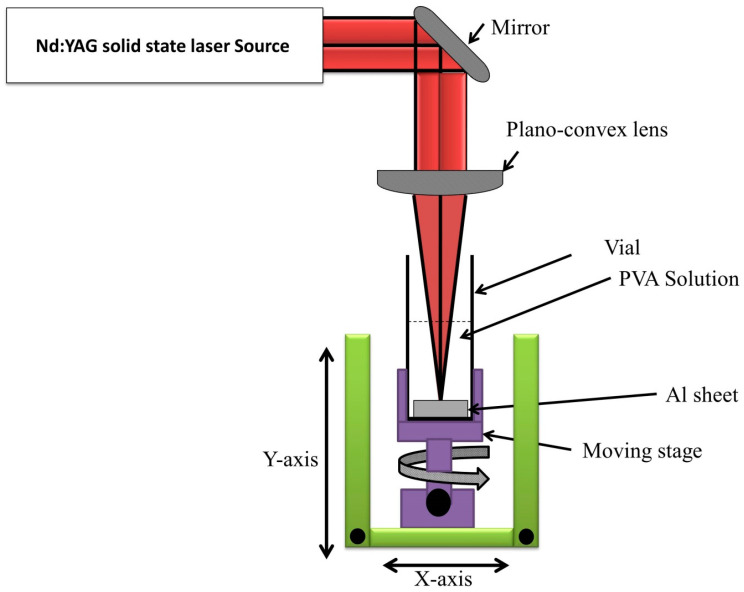
Schematic diagram of Al_2_O_3_-PVA nanocomposite by PLAL.

The Al_2_O_3_ nanostructured material was generated by the pulsed laser ablation of cleaned metal targets of Al sheet put in a glass vessel filled with 5 mL of the prepared PVA solution to form an Al_2_O_3_ nanostructured material embedding PVA. The laser ablation process produced by the laser beam of the first harmonic generation of nanosecond Nd:YAG laser, which produces a 10 Hz repetition rate and 150 mJ of laser energy. The ablation process was carried out by focusing the laser beam on the target’s surface with a planco-convex lens (70 mm) to produce a small spot that had a 20 µm diameter (Figure 1). The ablation procedure was carried out under mechanical stirring for 30 min to create distinct nanocomposite structures. Then, the prepared nanocomposite solution was separately cast in a glass Petri-dish to obtain the required films, followed by drying in glass Petri dishes with a thickness of about 2 mm in the form of free-standing and a diameter of 25 mm. The amount of immobilized Al_2_O_3_ NPs in PVA could be estimated in two ways: the first method was related to measuring the weight of the Al-sheet before and after the ablation process, followed by subtraction of the two values (weight _Al sheet_–weight _ablated Al sheet_) to estimate the amount of immobilized Al_2_O_3_ NPs in PVA. The second method was related to measuring the weight of PVA film before and after being embedded with Al_2_O_3_ NPs, followed by subtraction of the two values (weight _embedded PVA film_–weight _pure PVA film_) to estimate the amount of immobilized Al_2_O_3_ NPs in PVA. After using both methods and repeating each method three times, it was detected that the amount of embedded Al_2_O_3_ in the PVA film is 9.2 µg ± 0.051.

### 2.4. Investigation Techniques 

X-ray diffractometer (Shimadzu XRD 7000, Kyoto, Japan), UV–VIS-NIR spectrophotometer (JASCO 570 UV–VIS-NIR, Kyoto, Japan), atomic absorption spectroscopy (Shimadzu AA-6200; Shimadzu Co., Ltd., Kyoto, Japan), FT-IR (JASCO FT-IR 6100 spectrometer, Japan), and a scanning electron microscope associated with EDX analyzer detector (PHILIPS/FEI QUANTA 250, Prague, Czech Republic).

### 2.5. Adsorption Study

The batch sorption of Ni(II) ions onto a PVA-Al_2_O_3_ nanocomposite adsorbent in aqueous solutions at ambient temperature was examined. For that, a conical flask containing 100 mL of Ni^2+^ metal ion solution was filled with ultra-pure water and stirred to achieve an initial Ni solution concentration ranging from 10 to 100 mg/L before being put in a thermostatic shaker for absorption (200 rpm). The remaining Ni^2+^ ion concentration was then measured using atomic absorption spectroscopy. The following formulae were used to investigate the effect of various parameters on the adsorption capacity of Ni^2+^ [45,46].

Removal extent, percentage = (C_o_ − C_a_)/C_o_ × 100 (1)

where C_o_ and C_a_ are the concentrations of adsorbent material at the beginning of the adsorption process and at the equilibrium of the adsorption process, respectively.

## 3. Result and Discussion

### 3.1. Investigation of the Prepared Al_2_O_3_-PVA Nanocomposite

**Figure 2:**
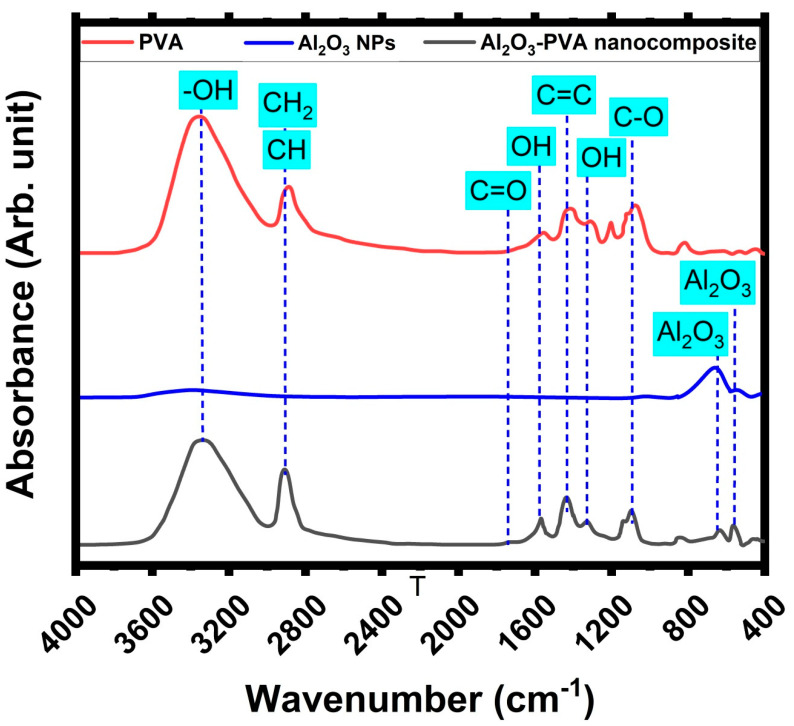
FT-IR spectra of PVA, Al_2_O_3_ nanoparticles, and Al_2_O_3_/PVA nanocomposite.

Figure 2 shows the FT-IR spectra of PVA, Al_2_O_3_ nanoparticles, and Al_2_O_3_-PVA nanocomposite to identify their vibrational absorption motion, which is produced by FT-IR spectra. The FT-IR spectra of pure PVA exhibited a strong and wide stretching band at 3334 cm^−1^ and a bending vibration at 1565 cm^−1^_,_ 1428 cm^−1^_,_ and 1325 cm^−1^ that can be attributed to the hydroxyl group because of intramolecular and intermolecular hydrogen bonding. Furthermore, stretching vibrational absorption bands at 2945 cm^−1^ and 2908 cm^−1^ appeared as a result of functional groups of the CH and −CH_2_ groups, respectively. Furthermore, the appearance of stretching vibration around 1100 cm^−1^ was related to the C-O functional group. These characteristics could be represented by the main functional groups of the PVA structure. In addition to the appearance of a vibrational stretching peak around 1739 cm^−1^ related to the C=O functional group, but with small intensity as it was related to the presence of leftover acetate groups after the hydrolysis of polyvinyl acetate to produce PVA [30,47,48]. In the case of the laser ablation of the Al sheet immersed in the ultra-pure water, the FT-IR spectra of Al_2_O_3_ nanoparticles exhibited two distinctive vibrational peaks of Al_2_O_3_, at 558 cm^−1^ and 632 cm^−1^, which are exhibited in the fingerprint region. Furthermore, the appearance of vibrational peaks characterized by a broad band stretching motion around 3342 cm^−1^ was related to the residual hydroxyl functional group (-OH) that was produced by atmospheric moisture. Moreover, there are no other peaks related to the residual organic compounds. In the case of Al_2_O_3_-PVA nanocomposite, the primary distinctive vibrational peaks of the PVA structure are still intact, but their strength and position have changed. The intensity of these hydroxyl group bands is clearly reduced, which is attributable to the interaction of the PVA structure with Al ions. Moreover, below 1000 cm^−1^, there are two distinct peaks of Al_2_O_3_, at 558 cm^−1^ and 632 cm^−1^, which are exhibited in the fingerprint area of the spectra as evidence for the incorporation of Al_2_O_3_ molecules in the polymer matrix. Moreover, it was clear that the strong electronegativity of the atoms formed from Al_2_O_3_ and injected into the polymeric matrix of PVA structure, which has a significant impact on the spectrum of adjacent group frequencies, may be responsible for the conformational changes in the width and intensity of the vibrational bands of PVA structure [20,41,49]. These significant fluctuations in the absorbance and frequencies of almost bands suggest that interactions between Al_2_O_3_ NPs and the polymeric structure of PVA, such as hydrogen bonds or van der Waals interactions, are forming between the (OH^−^)/(COO^−^) groups of PVA and the Al_2_O_3_ NPs. This was accomplished by reducing the crystallinity degree of nanocomposites and forming charge transfer complexes in which the Al_2_O_3_ NPs function as an electron acceptor and the PVA matrix works as an electron donor. These interactions alter the dynamics of the nanocomposite chain structure.

**Figure 3:**
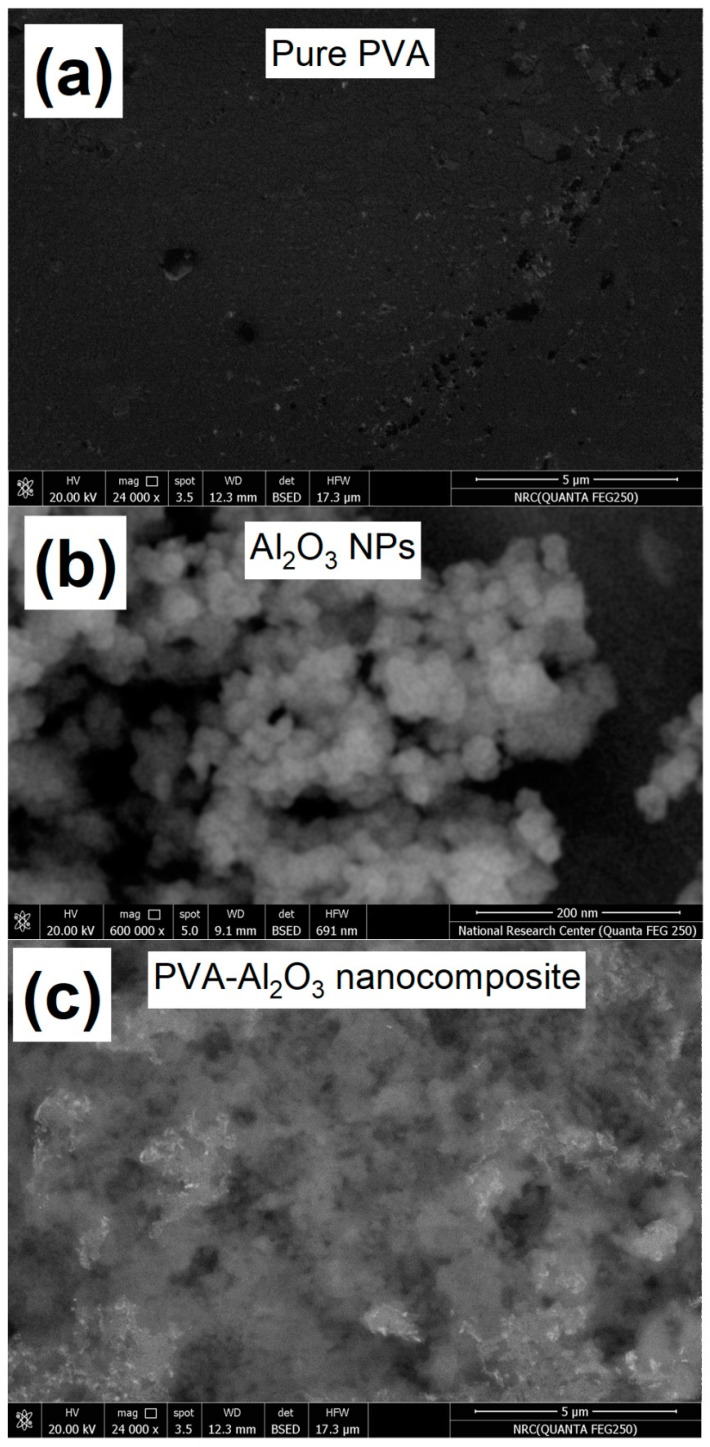
SEM image of (**a**) PVA, (**b**) Al_2_O_3_ nanoparticles, and (**c**) Al_2_O_3_/PVA nanocomposite.

FESEM was used to investigate the morphological changes in PVA caused by embedding with Al_2_O_3_ molecules as mentioned in Figure 3. The micrographs exhibit SEM of Al_2_O_3_ nanoparticles, PVA, and the hybrid with PVA-Al_2_O_3_ nanocomposite. The surface of the produced structure from the laser ablation of the Al sheet in the ultra-pure water showed a semi-spherical shape in the nanoscale form, while the SEM image of the PVA structure showed a uniform surface shape that reveals dispersed smooth microspores instead of uniform ones. Moreover, the surface of the polymer was significantly distorted during metal oxide contact, and the metal oxide particles were uniformly dispersed over the polymer surface compared to the pure PVA structure. As a result, the change in the surface of the PVA-Al_2_O_3_ nanocomposite reveals that the PVA surface was highly deformed, and the aluminum oxides were homogeneously distribution throughout the PVA surface.

**Figure 4:**
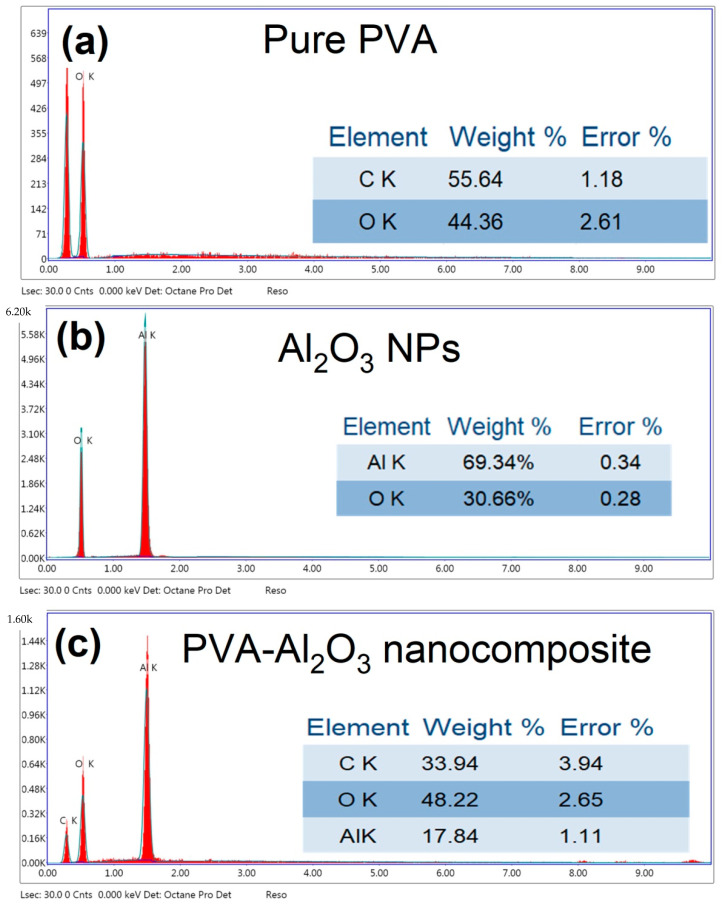
EDX elemental analysis of (**a**) PVA and (**b**) EDX elemental analysis of PVA before and (**c**) after embedding with Al_2_O_3_.

Furthermore, the elements on the surface of the PVA structure before and after embedding with Al_2_O_3_ NPs were examined using an EDX element analyzer. Figure 4 depicts the EDX spectra of a PVA-Al_2_O_3_ nanocomposite before and after Al_2_O_3_ NPs adsorption. This result verified the presence of a pure PVA structure as it was only comprised of the C and O elements, which represented the main constituted elements of the PVA structure. Moreover, the elemental analysis confirmed the formation of pure Al_2_O_3_ nanoparticles from the laser ablation process in the ultra-pure water as it was only comprised of the Al and O elements, which represented the main constituted elements of the Al_2_O_3_ structure. However, in the case of embedding with Al_2_O_3_, a new element (Al) was injected with C and O. So, the PVA structure was embedded with the Al element. Moreover, the phase of Al could be detected to be metallic or oxide form based on looking at the ratio of O/C in PVA structure before and after ablation of Al, which was 0.797 and 1.42, respectively. So, the amount of O was increased owing to the occurrence of Al in the oxide form (Al_2_O_3_).

**Figure 5:**
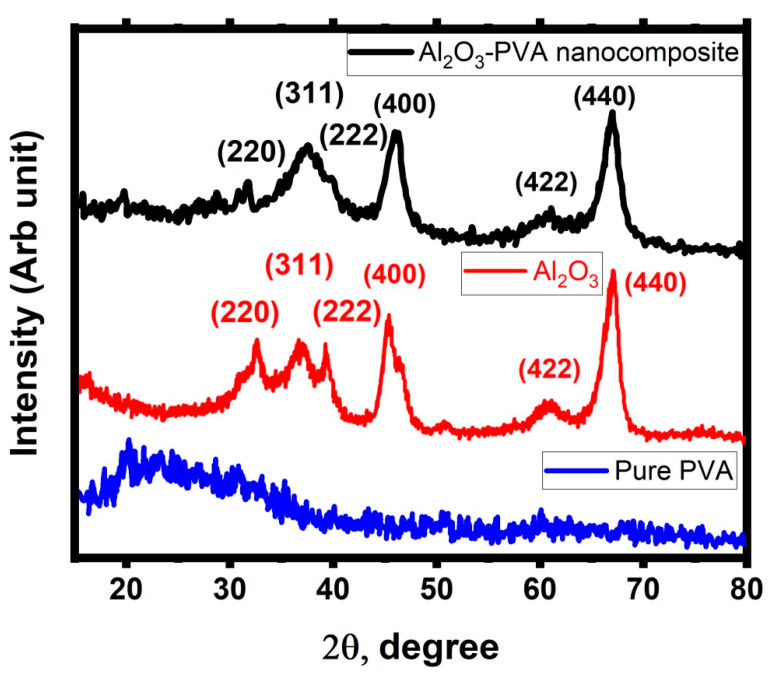
XRD diffractogram of PVA, Al_2_O_3_ nanoparticles, and Al_2_O_3_/PVA nanocomposite.

The XRD measurements were used to examine the crystallinity of the PVA structure with and without incorporated metal produced from the laser ablation of the Al sheet. Nanostructured materials were created by the PLAL method of an Al target immersed in different types of liquid media, and the resultant diffractogram spectra are given in Figure 5. In the case of pure PVA, there are two separate diffraction peaks at diffraction angles 2θ = 19.5° and 40.5°, which correspond to the indices planes (1 0 1) and (1 1 1), respectively [50]. Furthermore, no other peaks were observed related to any foreign impurities in the PVA structure.

In the case of the laser ablation of the Al sheet immersed in the ultra-pure water, the crystalline peaks appeared at 31.62°, 37.18°, 39.79°, 46.17°, 60.84°, and 67.13°, which correspond to (2 2 0), (3 1 1), (2 2 2), (4 0 0), (4 2 2), and (4 4 0), respectively, based on JCPD XRD card number 79-1558 of the cubic structure of Al_2_O_3_. Moreover, no other typical peaks were seen in the diffractogram, which may be produced from the other contaminants during the preparation steps. This observation confirmed the high purity of the produced nanostructure materials. Moreover, the main crystalline peak used to deetect the crystallite size of Al_2_O_3_ nanostructure using the Debye–scherrer equation [51].

(2) $$D=\frac{0.9\lambda }{\beta \cos\theta }$$

where *D*, λ, β, and *θ* are the crystallite size of Al_2_O_3_ nanostructure, used X-ray wavelength, FWHM of the diffraction peak at 2*θ* = 67.13°, and the Bragg angle (33.57°), respectively, its crystallite is 19.8 nm. In the case of laser ablation of Al sheet immersed in the PVA solution, the semi-crystalline structure was still present in the case of embedding with the generated nanostructured materials formed from the PLAL process of Al-target immersed in PVA solution, but the intensity was reduced by a large amount under the effect of the intermolecular hydrogen band produced between the PVA structure and nanostructured materials. Furthermore, the new crystalline peaks appeared at 31.62°, 37.18°, 39.79°, 46.17°, 60.84°, and 67.13°, which correspond to (2 2 0), (3 1 1), (2 2 2), (4 0 0), (4 2 2), and (4 4 0), respectively, based on JCPD XRD card number 79-1558 of the cubic structure of Al_2_O_3_ [52]. It was clear that the PVA diffraction peaks remained after being incorporated with Al_2_O_3_ NPs in the creation of a nanocomposite structure. It was demonstrated that the Al_2_O_3_ NPs incorporation had no effect on the distribution of the PVA’s crystallinity, while its area under the curve has changed to a lower value, indicating that the crystallinity of the PVA structure has reduced. This reduction could be related to the presence of intermolecular hydrogen bonding that occurred between its chain structure and Al_2_O_3_ NPs, leading to reducing the intermolecular interaction between the chains of PVA. Therefore, the electrostatic interaction of Al_2_O_3_ NPs with the PVA chain leads to disturbing the crystalline phase of the PVA structure. So, this observation could be represented as a confirmation of successful incorporation.

### 3.2. Adsorption Process

#### 3.2.1. Effect of pH

**Figure 6:**
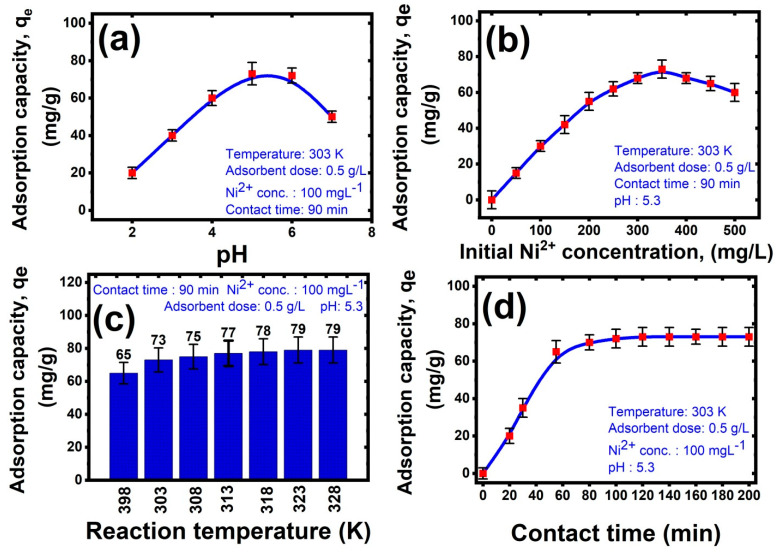
Effect of (**a**) pH, (**b**) beginning Ni^2+^ concentration, (**c**) reaction temperature, and (**d**) contact time on Ni(II) removal by Al_2_O_3_/PVA nanocomposite.

The pH of the solution is a critical variable in the adsorption process because it influences metal sorption via protonation and deprotonation of the adsorbent’s functional groups, the Ni speciation, adsorbent surface charge, and adsorbent ionization. As a result, the influence of pH on nickel sorption is explored in the pH range 2–7 for an initial concentration of 100 mg L^−1^ from Ni(II), an adsorbent dose of 0.5 g/L, and a temperature of study at 303 K as depicted in Figure 6a. This graph clearly shows that the adsorption capacity rises with a rising pH value, and the precipitation occurs in nickel solutions when the pH exceeds 7. So, the trials are not undertaken above a pH of 7. Furthermore, around pH 5.3, nickel adsorption capacity reaches a maximum of 62 mg g^−1^ and thereafter decreases with increasing pH levels [53,54,55,56]. This was related to:

1. At pH values higher than 6, the excess of alkaline –OH group has a greater tendency to combine with Ni^2+^ and form Ni(OH)_2_ participated, causing the adsorption to be diminished.
2. At pH values in the range 5–6, the surface charge of the hybrid nanocomposite turned to a negative charge due to the medium having a low acidic concentration, increasing the coordination between positively charged metal ions (Ni^2+^) and Al_2_O_3_-PVA nanocomposite by electrostatic attraction, leading to reach the maximum adsorption capacity. The results were consistent with Ni^2+^ adsorption on Al_2_O_3_-PVA nanocomposite as mentioned in the previous work [57].
3. At pH values lower than 5, the decrease in nickel adsorption capability at lower pH levels is related to protonation of the Al_2_O_3_-PVA nanocomposite by the acidic medium and the water molecules converted from H_2_O to H_3_O^+^, leading to a decrease in the number of charge carriers in the hybrid membrane for metal adsorption. In addition, competition for adsorption sites on the PVA structure created between H^+^ and Al^2+^ make an electrostatic repulsion between them [58,59,60,61].

#### 3.2.2. Effect of Initial Pollutant Concentration

The influence of initial Ni^2+^ concentrations on nanocomposite adsorption was examined from 0 mgL^−1^ to 500 mgL^−1^ of beginning Ni(II) concentration at a temperature of study at 303 K, pH 5.3, and an adsorbent dose of 0.5 g/L, and the findings are presented in Figure 6b. It was shown that the initial Ni^2+^ concentration in aqueous solution increased till it reached saturation and then decreased. So, the precision description of this process can be described as the percentage amount of Ni(II) removal greatly reduced as the beginning Ni(II) ion concentration content rises. This observation could be discussed when the initial Ni(II) ion concentration increased from 0 to 200 mg/L, the mass transfer of Ni(II) ions between the aqueous solution and the Al_2_O_3_ nanoparticles were supported, leading to improved interaction between Ni(II) ions and the Al_2_O_3_ nanoparticles and increasing the adsorption uptake of Ni(II). In other words, when the initial Ni(II) ion concentration was further increased from 200 to 500 mg/L, the adsorption capacity hit a plateau due to the strong binding sites on the Al_2_O_3_/PVA nanocomposites have been filled with the initial amounts of Ni(II) ions, which appeared from the adsorption isotherm analysis as the Langmuir and Freundlich models, which were compatible with the other scholars who had observed similar findings. So, from this study, the optimum beginning Ni(II) ion concentration for a good adsorption process was chosen before the plateau shape, at 100 mg/L as this value represented the highest rate of adsorption capacity and after this value, the rate of adsorption capacity started to be decreased [62,63,64].

#### 3.2.3. Effect of Temperature

Figure 6c depicts the influence of reaction temperature on the removal of Ni^2+^ ions for 100 mg L^−1^ beginning Ni(II) ion concentration, an adsorbent dose of 0.5 g/L, and pH of 5.3 for nickel solutions at varying temperatures from 298 K to 328 K. As the temperature rose from 298 to 328 K, the adsorption capacity of nickel ions grew from 65.8 mg g^−1^ to 87.4 mg g^−1^. These findings revealed that nickel ion sorption onto the Al_2_O_3_/PVA nanocomposite adsorbent was endothermic.

#### 3.2.4. Effect of Contact Time

Figure 6d depicts the influence of contact time on the removal of Ni^2+^ ions for starting metal concentrations of 0 and 500 mgL^−1^, an adsorbent dose of 0.1 g/L, an adsorbent dose of 0.5 g/L, and pH of 5.3 for nickel solutions at 303 K temperature. At varied starting metal concentrations of 0 and 100 ppm, the adsorption efficiency for nickel ions by the Al_2_O_3_/PVA nanocomposite rose dramatically and achieved equilibrium after 90 min. As a result, a time of 50 min was chosen as an equilibrium time for future tests. Furthermore, as demonstrated, the sorption capacity of Al_2_O_3_/PVA nanocomposite increases with time due to the increased diffusivity of nickel ions toward Al_2_O_3_/PVA nanocomposite [65].

### 3.3. Mechanism of Adsorption Process

**Figure 7:**
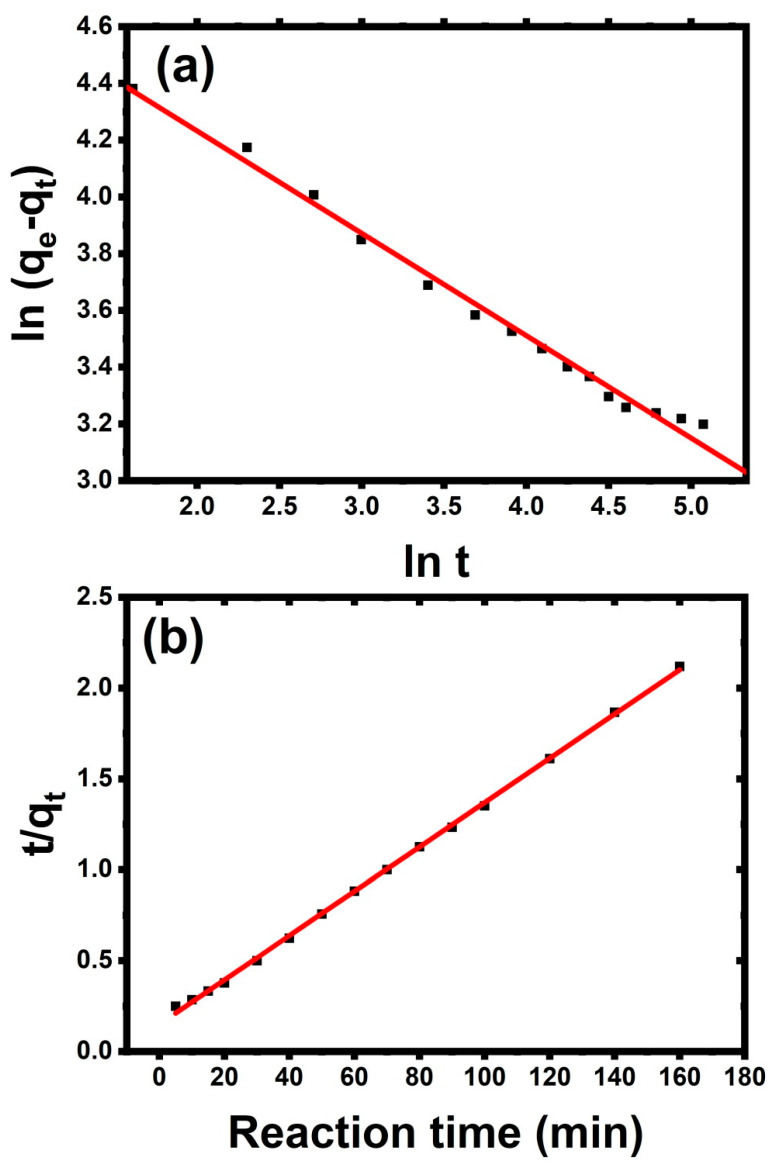
Adsorption kinetics of Ni(II) ions by Al_2_O_3_/PVA nanocomposite (**a**) Pseudo-first-order and (**b**) Pseudo-second-order.

The adsorption of metal ions of Ni^2+^ by the nanocomposite could be controlled in three ways. The first predicted mechanism was the nanocomposite adsorbent’s quick transfer to the external surface absorption of metal ions. The second predicted mechanism was the diffusion of metal ions via the pores of the nanocomposite. The third predicted mechanism might be linked to chemical adsorption between nanocomposite functional groups, such as hydroxyl groups, and metal ions. The experimental results were analyzed using pseudo-first-order or pseudo-second-order kinetic models to study the kinetic parameters of the adsorption of metal ions via the Al_2_O_3_/PVA nanocomposite [30,66,67,68].

(3) $$q_{t}=q_{e}\left(1-e^{-k_{1}t}\right)\;\mathrm{Pseudo}\text{-}\mathrm{first}\text{-}\mathrm{order}$$

(4) $$q_{t}=\left(k_{2}q_{e}{}^{2}t\right)/\left(1+q_{e}k_{2}t\right)\;\mathrm{Pseudo}\text{-}\mathrm{second}\text{-}\mathrm{order}$$

where $q_{t}$ is the amount of adsorbed metal after *t * time, $q_{e}$ is the amount of adsorbed metal after equilibrium time, $k_{1}$ is the constant of pseudo-first-order, and $k_{2}$ is the constant of pseudo-second-order. From Figure 7, the intercept, slope, and the correlation coefficient of Pseudo-first-order are 4.95347 ± 0.03878, −0.3606 ± 0.00983, and 0.98969, respectively, while the intercept, slope, and the correlation coefficient of Pseudo-second-order are 0.15013 ± 0.00719, 0.01219 ± 8.87805 × 10^−5^, and 0.99926, respectively. So, the value of $k_{1}$ and $k_{2}$ are 0.3606 and 9.9 × 10^−^^4^ gmg^−1^ min^−1^, respectively. Therefore, in the case of the pseudo second-order, the predicted equilibrium adsorption capacity was more compatible with its experimental value.

## 4. Conclusions

A novel hybrid PVA/Al_2_O_3_ nanocomposite was created in this study using a simple green potential approach. This approach used a pulsed laser ablation method to create a hybrid nanocomposite from an Al sheet immersed in PVA solution. The successful embedding of PVA structure with Al_2_O_3_ nanoparticles was demonstrated by FTIR, XRD, and SEM-EDAX investigation of PVA before and after contact with Al_2_O_3_ nanoparticles. It can be utilized as a cost-effective alternative to activated carbons, membrane filtration, and ion-exchange adsorbents. The adsorbent effectively removes Ni(II) from aqueous solutions, with even greater removal achieved at low concentrations of about 100 mg/L. The adsorption was shown to be pH-dependent, with pH 5.3 yielding the most clearance. The pseudo-second-order rate kinetics was used to manage the removal process. The adsorption findings revealed that hybrid PVA/Al_2_O_3_ nanocomposite may be utilized to efficiently remove Ni(II) from aqueous solutions.

## Figures and Tables

**Table 1:** Recent survey of nanocomposite based on Al_2_O_3_-PVA for removal heavy metals or degradation of organic pollutants.

Composite	Organic Pollutants/Heavy Metals	Efficacy
Al_2_O_3_-PVA [42]	phosphate	95%
PVA–ZnO–Al2O_3_ [43]	MB	100%
Polythiophene/PVA/Al_2_O_3_ [44]	Pb(II),	97.3%
	Zn(II),	89.4%
	Cd(II)	95.8%
Polyaniline/PVA/Al_2_O_3_ [44]	Pb(II),	89.78%
	Zn(II),	84.9%
	Cd(II)	79.2%

## Data Availability

Not Applicable.
